# Supplementary material for: Influenza A Virus Infection Reactivates Human Endogenous Retroviruses Associated with Modulation of Antiviral Immunity
Source: Viruses. 2022 Jul 21;14(7):1591. doi: 10.3390/v14071591 (PMC9320126; doi:10.3390/v14071591)
Supplement: Supplementary file 1 [file viruses-14-01591-s001.zip › MotifAnalysis.zip!/MotifOutput_PR8M/homerResults/motif13.similar.html]

motif13

## Information for motif13

A
C
G
T
A
C
G
T
A
C
T
G
A
C
T
G
C
G
T
A
A
C
T
G
C
G
T
A
C
T
G
A
A
G
T
C
A
G
T
C
A
C
G
T
A
C
G
T
  
Reverse Opposite:  

C
G
T
A
C
G
T
A
A
C
T
G
C
T
A
G
A
G
C
T
A
C
G
T
A
G
T
C
A
C
G
T
A
G
T
C
A
G
T
C
C
G
T
A
G
T
C
A
  

|  |  |
| --- | --- |
| p-value: | 1e-12 |
| log p-value: | -2.945e+01 |
| Information Content per bp: | 1.966 |
| Number of Target Sequences with motif | 8.0 |
| Percentage of Target Sequences with motif | 34.78% |
| Number of Background Sequences with motif | 226.8 |
| Percentage of Background Sequences with motif | 0.50% |
| Average Position of motif in Targets | 92.0 +/- 56.0bp |
| Average Position of motif in Background | 104.6 +/- 87.4bp |
| Strand Bias (log2 ratio + to - strand density) | 10.0 |
| Multiplicity (# of sites on avg that occur together) | 1.25 |
| Motif File: | file (matrix) reverse opposite |

### Similar de novo motifs found

|  |  |  |  |  |  |  |  |
| --- | --- | --- | --- | --- | --- | --- | --- |
| Rank | Match Score | Redundant Motif | P-value | log P-value | % of Targets | % of Background | Motif file |
| 1 | 0.613 | C A T G C G T A A C G T A C G T C G T A A C T G C G T A A C T G C G T A C T G A | 1e-12 | -28.842476 | 26.09% | 0.12% | motif file (matrix) |
| 2 | 0.714 | A C T G C G A T C A G T G T A C C G A T G A C T T G A C C G T A | 1e-11 | -27.079400 | 47.83% | 2.42% | motif file (matrix) |
| 3 | 0.890 | A C G T A C T G C T A G C G T A T A C G C G T A C T G A A G T C A T G C A C G T | 1e-10 | -23.844289 | 26.09% | 0.28% | motif file (matrix) |
| 4 | 0.628 | A C G T G T A C C T G A C T G A A C T G A C T G A C G T A C G T | 1e-9 | -21.383309 | 34.78% | 1.37% | motif file (matrix) |
